# Could the Extended Phenotype Extend to the Cellular and Subcellular Levels in Insect-Induced Galls?

**DOI:** 10.1371/journal.pone.0129331

**Published:** 2015-06-08

**Authors:** Renê Gonçalves da Silva Carneiro, Priscilla Pacheco, Rosy Mary dos Santos Isaias

**Affiliations:** Universidade Federal de Minas Gerais, Instituto de Ciências Biológicas, Belo Horizonte, Minas Gerais, Brazil; University of Manitoba, CANADA

## Abstract

Neo-ontogenesis of plant galls involves redifferentiation of host plant tissues to express new phenotypes, when new cell properties are established via structural-functional remodeling. Herein, *Psidium cattleianum* leaves and *Nothotrioza cattleiani* galls are analyzed by developmental anatomy, cytometry and immunocytochemistry of cell walls. We address hypothesis-driven questions concerning the organogenesis of globoid galls in the association of *P*. *cattleianum - N*. *cattleianum*, and *P*. *myrtoides - N*. *myrtoidis*. These double co-generic systems represent good models for comparing final gall shapes and cell lineages functionalities under the perspective of convergent plant-dependent or divergent insect-induced characteristics. Gall induction, and growth and development are similar in both galls, but homologous cell lineages exhibit divergent degrees of cell hypertrophy and directions of elongation. Median cortical cells in *P*. *cattleianum* galls hypertrophy the most, while in *P*. *myrtoides* galls there is a centrifugal gradient of cell hypertrophy. Cortical cells in *P*. *cattleianum* galls tend to anisotropy, while *P*. *myrtoidis* galls have isotropically hypertrophied cells. Immunocytochemistry evidences the chemical identity and functional traits of cell lineages: epidermal cells walls have homogalacturonans (HGAs) and galactans, which confer rigidity to sites of enhanced cell division; oil gland cell walls have arabinogalactan proteins (AGPs) that help avoiding cell death; and parenchyma cell walls have HGAs, galactans and arabinans, which confer porosity. Variations in such chemical identities are related to specific sites of hypertrophy. Even though the double co-generic models have the same macroscopic phenotype, the globoid morphotype, current analyses indicate that the extended phenotype of *N*. *cattleiani* is substantiated by cellular and subcellular specificities.

## Introduction

The ontogenesis of plant organs occurs via conservative cellular mechanisms that act synergistically for the determination of the variable forms and functions observed in nature [1]. Plant galls represent the neo-ontogenesis [2] of their host organs towards a new phenotype, i. e., the gall morphotypes [3]. For the generation of these gall morphotypes, plant tissues and cells respond to the stimuli of gall-inducing herbivores by redifferentiating new cell types [4]. In the context of gall structure, such cells have adaptive significance for the gall inducers as far as their protection and nourishment are concerned [5, 6].

Neotropical gall morphotypes have been studied on developmental anatomy basis, focusing on the responses of tissue hyperplasia and cell hypertrophy, the degree of isotropy and/or anisotropy of cell expansion [7], and the structural-functional traits derived from these responses [8– 12]. More recently, the immunocytochemistry of cell walls in gall tissues have been studied [13, 14], and this helped in elucidating the functionalities of the cell walls, and their roles in gall development. Under the perspective of the developmental anatomy and immunocytochemistry of plant cell walls, Carneiro et al. [2] provided an interesting insight into the organogenesis of a globoid leaf gall on *Psidium myrtoides* (Myrtaceae) induced by *Nothotrioza myrtoidis* (Triozidae). The composition of cell walls during the development of this gall influences dynamic properties of cell lineages in terms of rigidity, flexibility, porosity, and adhesion, as described for plant organs in general [15, 16, 17]. Such properties affected the mechanisms of cell growth, i. e., division and/or expansion, and determined the establishment of a centrifugal gradient of cell hypertrophy with isotropic growth in the cortex of *N*. *myrtoidis* galls [2].

Current model of study, the interaction between the host plant *Psidium cattleianum* Sabine (Myrtaceae) and the gall-inducing herbivore *Nothotrioza cattleiani* Burckhardt (Triozidae) results in the morphogenesis of globoid galls, very similar to those of the double co-generic system, *P*. *myrtoides-N*. *myrtoidis* [2]. The galls on *P*. *cattleianum* and *P*. *myrtoides* are both globoid [3], protrude to the abaxial surface of the leaf lamina, and have univoltine cycles [18, 19]. To the extent of ecological and macro-morphological aspects, the phenotypic expression of the genes from the two species of *Nothotrioza* exerts biochemical influence on the cells of two species of *Psidium*. Unexpectedly, they generate the same extended phenotype [20], the globoid gall morphotype. As the gall structure is adaptive for their inducers [21], and varies according to the phylogeny of galling insects [22], we hypothesize that *Nothotrioza* spp. galls on *Psidium* spp. are unique entities, i. e., true extended phenotypes with species-specific traits at the cellular and subcellular levels. The following questions are addressed: (1) Are there divergent patterns on the way *Nothotrioza* spp. manipulate the standard leaf morphogenesis of *Psidium* spp. towards the ontogenesis of globoid galls? (2) Should the gradients of cell transformations be quantitatively divergent on the co-generic systems? (3) Is the distribution of pectins and cell wall proteins a conservative trait of the cell lineages within and between the *Nothotrioza* spp. galls?

## Material and Methods

### Study area

The studied population of *P*. *cattleianum* is located at the Parque Estadual Pico do Marumbi, municipality of Piraquara, state of Paraná, Brazil. Individuals (n = 5) with galled leaves were marked and sampled during 2012 and 2013.

### Ethics statement

The authors declare that the studied species are not protected and/or threatened. The access to the protected area of the Parque Estadual Pico do Marumbi, and the permission for field sampling were granted by the Instituto Ambiental do Paraná —IAP (document number 34.14), and by the Instituto Chico Mendes de Conservação da Biodiversidade—ICMBio (document number 33424–4).

### Structural analyses

Samples of young and mature leaves, and galls at the stages of induction, growth and development, maturation and senescence [19] (n = 5 per developmental stage) were collected from different individuals, and fixed in Karnovsky’s solution in 0.1 M phosphate buffer (pH 7.2) [23]. The material was dehydrated in ethanol series [24], embedded in glycolmethacrylate (Leica, Wetzlar, Germany), sectioned (6–10 μm) with a rotary microtome Hyrax (Zeiss, Oberkochen, Germany), stained with 0.05% Toluidine O blue, pH 4.6 [25]. Part of the material was dehydrated in n-butyl series [24], embedded in Paraplast [26], sectioned (12–14 μm) with a rotary microtome (Jung biocut) and stained with 9:1 (v/v) Astra blue and safranin [27] (modified to 0.5%). Histological slides were observed and photographed under light microscope (Leica DM500) coupled with digital camera (Leica ICC50 HD).

### Cytometry and histometry

Digital images were obtained with an optical photomicroscope (Leica DM500), and analyzed with the AxioVision software, Zeiss Imaging Systems, version 4.7.2 [28]. Cells from the adaxial and abaxial protoderm, the adaxial, median and abaxial layers of the ground meristem of non-galled leaves, as well as their redifferentiated gall tissues had their areas measured individually (n = 50; 5 cells of each tissue per image, 10 images per developmental stage). In addition, the major axes of these cells—anticlinal and periclinal—were measured in each tissue lineage and the ratio between them was used as indicator of the degree of isotropy/anisotropy. The thickness of the mesophyll was measured at different developmental stages.

### Immunocytochemistry of plant cell wall epitopes

Unstained material (n = 3; from different individuals) fixed in Karnovsky’s solution in 0.1 M phosphate buffer (pH 7.2) [23], dehydrated in ethanol series [24], and embedded in glycolmethacrylate (Leica) was incubated with the monoclonal antibodies JIM5, JIM7, LM1, LM2, LM5, LM6, LM19 and LM20 (Centre for Plant Sciences, University of Leeds, UK). These antibodies specifically bind epitopes of low methylesterified homogalacturonans (HGAs) (JIM5) [15, 16, 29, 30], medium- high methylesterified HGAs (JIM7) [15, 16, 30], extensins (LM1) [31–34], arabinogalactan proteins (APGs) (LM2) [35], galactans (LM5) [36], arabinans (LM6) [37], unesterified HGAs (LM19), and high methylesterified HGAs (LM20) [38]. The sections were immersed in blocking solution with 3% (w/v) powdered milk in PBS for 30 min to avoid cross labelling, and incubated with primary antibodies in PBS for 2 h at room temperature. For the control tests, the primary antibodies were suppressed. Sections were washed in PBS, and incubated in the secondary antibody anti-rat IgG—FITC (Sigma, St. Louis, MO, USA) in PBS for 2 h in the dark. After washing in PBS, the sections were mounted in 50% glycerin and analyzed using a Confocal Zeiss 510 META microscope, with excitation wavelength of 488 ηm and 505–530 ηm emission filter.

### Statistical analyses

Statistical analyses of the cytometric values were performed using the software Past 3 [39]. Normal data (Shapiro-Wilk test) were compared by parametric tests of ANOVA followed by t-test or multiple tests of Tukey. Non-normal data were compared using the non-parametric tests of Kruskal-Wallis followed by Dunn's multiple tests. All tests used α ≤0.05.

## Results

### Ontogeny of non-galled leaves and galls

#### Non-galled leaves

**Fig 1:**
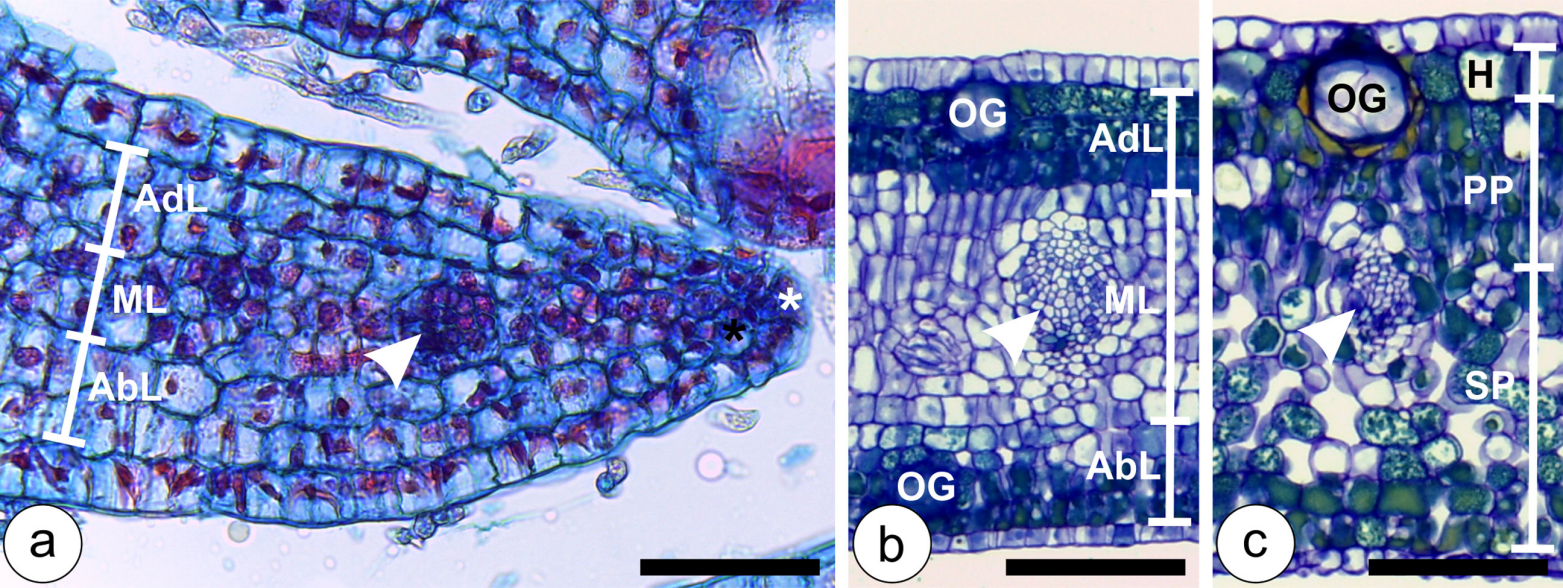
Developmental anatomy of *Psidium cattleianum* leaves. (**a**) Early developmental stage of the leaf, with uniseriate epidermis derived from marginal initial (white asterisk), ground meristem derived from submarginal initial (black asterisk) divided into two adaxial layers (AdL), two median layers (ML), and two abaxial layers (AbL). Procambium (arrow head) differentiates from the median layers of the ground meristem. (**b**) Young leaf with uniseriate epidermis, three-layered AdL, six-layered ML, three-layered AbL, and collateral vascular bundles (arrow head). Developing oil glands (OG) differentiate both from the adaxial and abaxial epidermal surfaces. (**c**) Mature leaves with uniseriate epidermis, one-layered hypodermis (H), three-layered palisade parenchyma (PP) and eight-layered spongy parenchyma (SP). Mature vascular bundles (arrow head) and oil glands (OG) are observed. Bars: (**a**) 50 μm; (**b, c**) 100 μm.

The leaves of *P*. *cattleianum* are simple, opposite, and emerge in pairs from the apical meristem. The uniseriate protoderm is derived from the divisions of the marginal initials, and the multilayered ground meristem is derived from the submarginal initials. At the early stages of leaf development, the ground meristem is divided into three regions, i.e. adaxial, median and abaxial layers. The cells of such layers divide anticlinally causing the elongation of the limb, and periclinally, increasing mesophyll thickness. Initially, the ground meristem has two adaxial layers, two median layers and two abaxial layers. Procambial strands differentiate from the median layers (Fig 1a). As the leaves develop, periclinal divisions add one cell layer both to the adaxial and abaxial layers, which end up with three layers each, and have phenolic inclusions. The median layers, whose cells have hyaline protoplasts, divide periclinally, reaching six cell layers (Fig 1b). Mature leaves have uniseriate epidermis, 12-layered mesophyll with one-layered hypodermis derived from the uppermost cell layer of the ground meristem. The palisade parenchyma is derived from the adaxial and the uppermost cell layers of the median ground meristem. The spongy parenchyma is 8 to 9-layered and is derived from both the median and abaxial layers of the ground meristem (Fig 1c). Vascular bundles are collateral and often surrounded by a sclerenchymatic bundle sheath.

#### Galls

**Fig 2:**
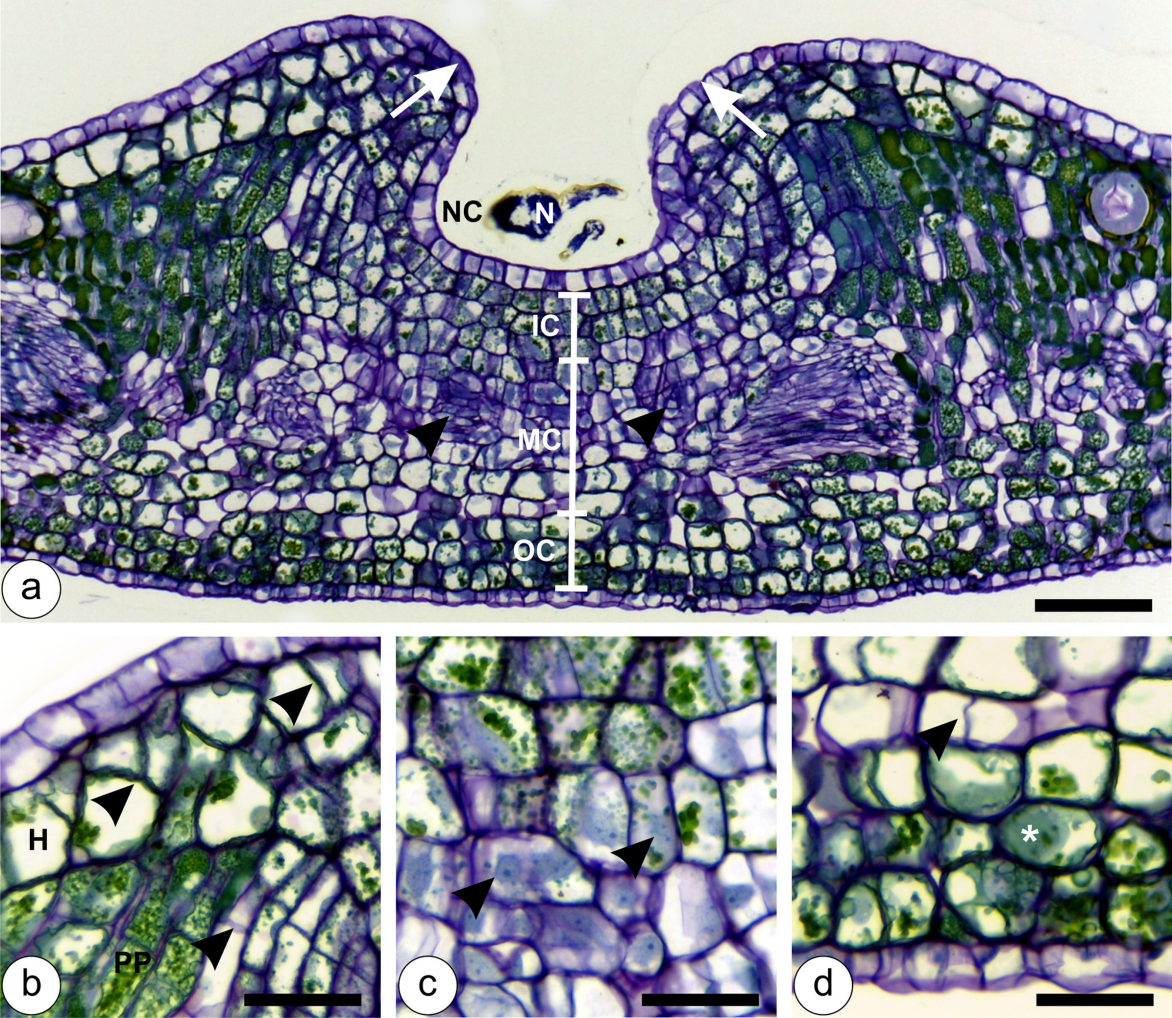
Developmental anatomy of *Nothotrioza cattleiani* galls at the stage of induction. (**a**) General view, showing the growth of projections (arrows) over the first instar nymph (N) to form the nymphal chamber (NC). Gall cortex has three-layered inner cortex (IC), six-layered median cortex (MC) and three-layered outer cortex (OC), and redifferentiated procambium strands (arrow heads). (**b**) Detail of the projections with sites of tissue hyperplasia (arrow heads) in the hypodermis (H) and palisade parenchyma (PP). (**c**) Detail of median cortical cells with hyaline protoplasts and conspicuous nuclei and nucleoli. (**d**) Detail of outer cortical cells with slightly hypertrophied cells, anticlinal cell divisions (arrow head) and phenolic inclusions in the protoplast (asterisk). Bars: (**a**) 100 μm; (**b-d**) 50 μm.

*N*. *cattleiani* induces galls on young leaves of *P*. *cattleianum*, which maintain its tissue zonation (Fig 2a). The inner and the outer gall epidermises are derived from the adaxial and abaxial leaf epidermises, respectively. Oil glands differentiate exclusively from the gall outer epidermal cells and are localized among the outermost cortical cell layers. The cortex has three zones: inner, median and outer layers, which redifferentiate from the adaxial, median and abaxial layers of the ground meristem of the young leaves, respectively.

Gall induction occurs via the stimuli of the first instar nymphs, which establish on the adaxial leaf surface, and start feeding preferably on the vascular bundles. Projections on the adaxial leaf surface grow around the insect body, and constitute the first set of anatomical alterations towards the development of the galls (Fig 2a and 2b). The epidermal cells divide anticlinally and elongate periclinally, while the cells of the adaxial layers divide both periclinally and anticlinally for the morphogenesis of the projections (Fig 2b). Such projections grow over the insect, towards one another to form an occluded ostiole; they enclose the insect within a nymphal chamber, determining the endophytic habitus of *N*. *cattleiani*. Right below the nymphal chamber, the gall cortex has slightly hypertrophied cells that divide anticlinally (Fig 2c and 2d; arrows). The median cortical layers have hyaline protoplasts, large nuclei and conspicuous nucleoli (Fig 2c). The inner and outer layers have inconspicuous nuclei and phenolic inclusions in the vacuoles (Fig 2c and 2d). The outer gall epidermis remains uniseriate, as its cells divide anticlinally (Fig 2d).

**Fig 3:**
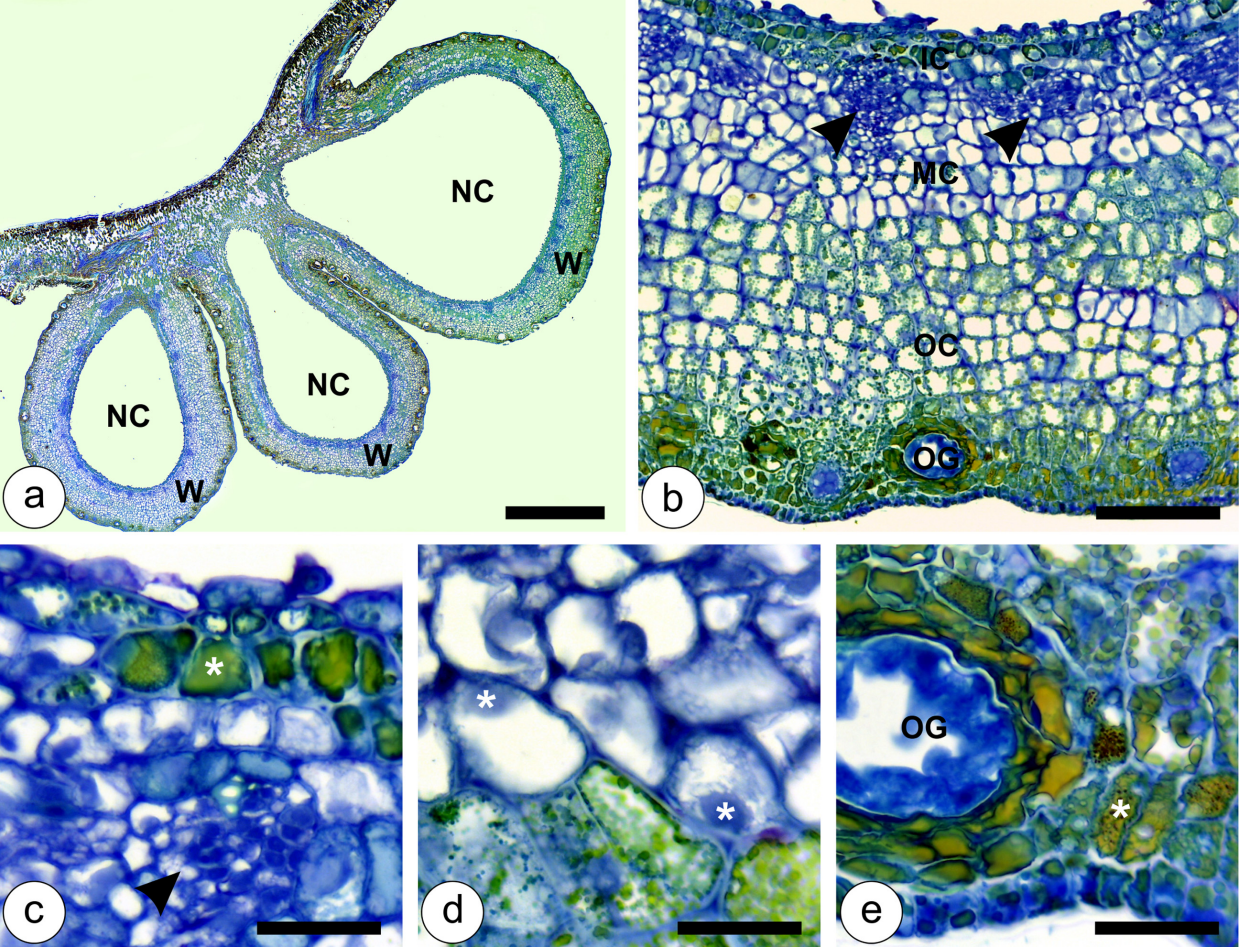
Developmental anatomy of *Nothotrioza cattleiani* galls at the stage of growth and development. (**a**) General view of three galls, with thin walls (W) and ample nymphal chamber (NC). (**b**) Detail of gall cortex with reduced inner cortex (IC), and hyperplasic median (MC) and outer (OC) cortices. Vascular bundles (arrow heads) are distributed closest to the nymphal chamber in the MC, and the oil glands (OG) differentiate exclusively from to outer epidermis in the OC. (**c**) Detail of the uniseriate inner epidermis, inner cortical cells with phenolic inclusions (asterisk), and vascular bundles with few tracheal elements, and abundant phloem and parenchyma (arrow head). (**d**) Detail of the median cortical cells with hyaline protoplasts and conspicuous nuclei (asterisks). (**e**) Detail of outer cortical cells with phenolic inclusions (asterisk). Oil glands are fully developed. Bars: (**a**) 2 mm; (**b**) 200 μm; (**c-e**) 50 μm.

**Fig 4:**
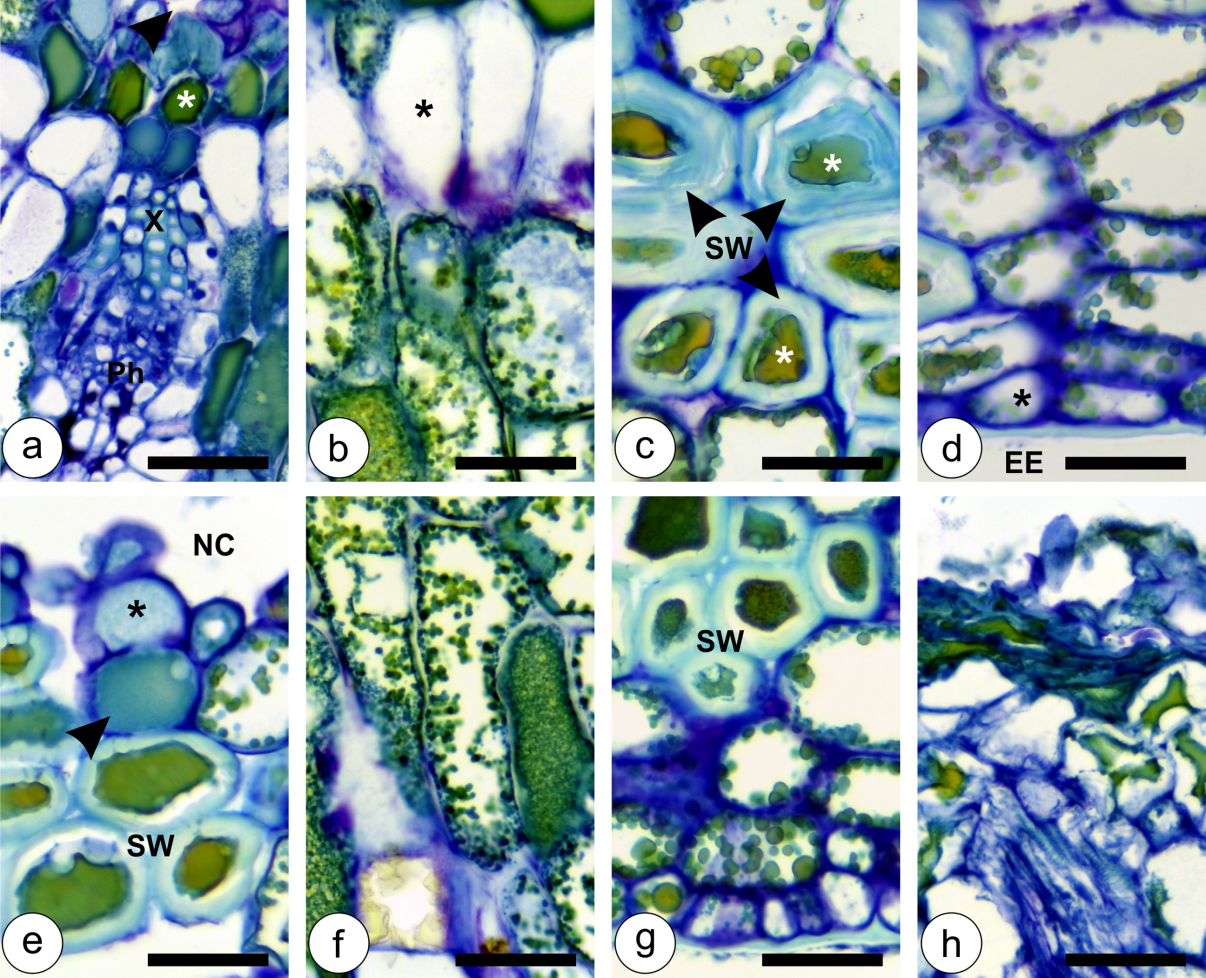
Developmental anatomy of *Nothotrioza cattleiani* galls. (**a-d**) Stage of maturation. (**e-h**) Stage of senescence. (**a**) Detail of hypertrophied inner epidermal cells lining the nymphal chamber (arrow head), inner cortical cells with phenolic inclusions (asterisk) and collateral vascular bundles with equal portions of xylem (X) and phloem (Ph). (**b**) Detail of hypertrophied and anticlinally elongated median cortical cells with hyaline protoplasts (asterisks), and the outer cortical cells with phenolic inclusions. (**c**) Detail of live sclereids at the middle of the outer cortex, with phenolic inclusions and thick secondary walls (SW). (**d**) Detail of the periclinally elongated outermost cortical cell layers adjacent to the outer epidermis (asterisk) in contact with the external environment (EE). (**e**) Detail of inner epidermal (asterisk) lining the nymphal chamber (NC), and inner cortical cells (arrow head); median cortical cells with secondary lignified cell walls (SW) and phenolic inclusions. (**f**) Detail of phenolic substances accumulated in the anticlinally elongated median cortical cells (asterisk). (**g**) Detail of the outer cortical cells and outer epidermis with secondary walls (SW) and vacuolated non-lignified cells. (**h**) Detail of necrotic sites in the gall apex that lead to gall dehiscence. Bars: 50 μm.

During the stage of growth and development, when the second, third and fourth instar nymphs are inside the galls, the second set of anatomical alterations takes place. At this stage, the gall cortex enlarges together with the nymphal chamber for the determination of the gall globoid shape (Fig 3a). The inner cortical layers divide mainly anticlinally, so the number of cell layers does not increase. The median and outer cortical layers, on the other hand, divide both anticlinally and periclinally, and are responsible for increasing the number of gall cortical cell layers. Vascular bundles redifferentiate from the middle layers of the gall cortex, close to the nymphal chamber (Fig 3b). The inner and the outer epidermal layers remain uniseriate, and divide anticlinally to accompany the growth of the developing gall. The inner epidermal cells divide sparsely and elongate periclinally, while the outer epidermal cells divide intensely, and do not elongate (Fig 3c and 3e). Cells of the inner and the outer cortical layers remain with phenolic inclusions (Fig 3c and 3e), while the cells of the median cortical layers have hyaline protoplasts (Fig 3c and 3d). Vascular bundles are collateral, with few tracheal elements, and abundant phloem and parenchyma (Fig 3c). The galls increase in size until the stage of maturation, which begins when the nymphs reach the fifth instar, and lasts until they molt into adults, and are ready to leave the galls. The structural organization in the cortex of mature galls is similar to that of the previous developmental stage, with hypertrophied cells throughout the cortex (Fig 4a–4d). The inner epidermis remains uniseriate; the inner cortical cells have phenolic inclusions, and the vascular bundles develop into a collateral arrangement, with equal portions of xylem and phloem (Fig 4a). In the transition between the median cortical cells, which have hyaline protoplasts, and the outer cortical cells with phenolic inclusions, it is possible to observe anticlinally elongated cells (Fig 4b). Sclereids (5–6 layers) differentiate from the median layers of the outer cortical cells, and constitute the most remarkable anatomical feature of mature galls (Fig 4c). The outermost cortical cells located between the sclerenchyma layers and the outer epidermis are vacuolated and periclinally elongated (Fig 4d).

At their final developmental stage, senescent galls present anatomical alterations, which lead to a dehiscence mechanism by which the galls open. Median cortical cells lignify, but remain live with phenolic inclusions within their protoplasts (Fig 4e). Phenolic substances accumulate gradually in the median cortical cells, which are elongated (Fig 4f). The outer cortical cells and outer epidermis remain similar to the previous developmental stage, with sclereids and vacuolated non-lignified cells (Fig 4g). Ultimately, necrotic sites develop from the apex towards gall base, and from the innermost towards the outermost cortical cell layers (Fig 4h). The necrotic tissues crack due to the tension exerted by the adjacent live and turgid cells, thus rupturing the gall wall, and allowing the scape of the adult insect.

### Dynamics of cell sizes and shapes

During the maturation of the non-galled leaves of *P*. *cattleianum*, cells from all tissues grew. Epidermal cells on the adaxial and abaxial surfaces of mature leaves increase in area approximately 100% and 90% in relation to the young leaves, respectively. Chlorophyllous parenchyma cells from the adaxial, median and abaxial layers of the young leaves grew approximately 130% towards the stage of leaf maturation (Table 1). As the galls are induced on young leaves, the tissues undergo different dynamics of cell size and shape. The inner epidermal cells derived from the adaxial epidermis of the leaves grew 25% towards the stage of gall induction, 120% towards the stage of growth and development, 72% towards the stage of maturation, and 20% towards the stage of senescence. Parenchyma cells of the inner cortical layers derived from the adaxial cell layers of leaves grew 17% towards the stage of gall induction, 180% towards the stage of growth and development, 60% towards the stage of maturation, and 80% towards the stage of senescence. The median cortical layers derived from the median layers of the leaves grew 24% towards the stage of gall induction, 395% towards the stage of growth and development, 44% towards the stage of maturation, and 23% towards the stage of senescence. The outer cortical cells derived from the abaxial cell layers of the leaves grew 69% towards the stage of gall induction, 148% towards the stage of growth and development, 138% towards the stage of maturation, and decrease their size in about 12% towards the stage of senescence. The outer epidermal cells derived from the abaxial epidermis of the leaves grew 36% towards the stage of gall induction, 100% towards the stage of growth and development, 6% towards the stage of maturation, and decrease their size in about 22% towards the stage of senescence (Table 1). The thickness of the mesophyll of young leaves (345.06 ± 12.55 μm) increases towards mature leaves (447.64 ± 5.77 μm). The mesophyll is slightly thinner in the galls at the induction stage (334.19 ± 12.22 μm) when compared to young leaves. As the galls developed, mesophyll thickness increased massively at the stages of growth and development (873.14 ± 82.50 μm), maturation (1086.42 ± 31.75 μm) and senescence (1170.59 ± 43.92 μm).

**Table 1:** Dynamics of cell hypertrophy during the ontogenesis of *Psidium cattleianum* leaves and *Nothotrioza cattleiani* galls (mean cell area- μm² ± standard deviation).

	Cell lineages				
Developmental stages	Adaxial/inner epidermis	Adaxial meristem/ inner cortex	Median meristem/ median cortex	Abaxial meristem/ outer cortex	Abaxial/outer epidermis
Young non-galled leaf	254.96 ± 10.18*f*	324.57 ± 12.94*d*	336.53 ± 13.32*e*	236.95 ± 8.70*d*	149.22 ± 6.45*d*
Mature non-galled leaf	510.63 ± 15.64*ed*	768.37 ± 26.97*c*	764.61 ± 28.49*d*	549.10 ± 25.07*d*	288.57 ± 10.42*c*
Gall induction	319.01 ± 20.37*d*	381.00 ± 27.56*d*	417.99 ± 24.38*de*	399.03 ± 31.09*d*	203.51 ± 12.45*cd*
Gall growth and development	705.05 ± 46.88*ce*	1077.00 ± 65.12*c*	2066.94 ± 122.63*c*	993.39 ± 78.59*c*	406.62 ± 34.59*b*
Gall maturation	1216.55 ± 67.23*b*	1735.63 ± 84.40*b*	2993.51 ± 119.72*b*	2372.67 ± 152.36*a*	434.92 ± 22.46*a*
Gall senescence	1469.29 ± 115.32*a*	3135.36 ± 159.55*a*	3695.63 ± 161.63*a*	2091.96 ± 98.98*b*	340.91 ± 19.52*bc*

Values followed by different italic letters in the columns indicate significant difference between the cell areas from the different lineages at different developmental stages. (ANOVA followed by t-test or multiple tests of Tukey; Kruskal-Wallis followed by Dunn's multiple tests. α = 0.05).

The adaxial epidermal cells are anticlinally elongated in the young leaves, and elongate periclinally in mature leaves. During gall development on young leaves, the adaxial epidermal cells originate the anticlinally elongated inner epidermal cells at gall induction. Such cells elongate periclinally at the stages of growth and development, and maturation, and tend to grow isotropically at gall senescence (Table 2). The cells of the adaxial meristem in the young leaves are slightly anticlinally elongated. During the differentiation of palisade parenchyma, this pattern is maintained during leaf maturation. The inner cortical cells derived from the adaxial meristem are anticlinally elongated, tending to grow isotropically in the galls at growth and developmental stage. During gall maturation and senescence, these cells elongated anticlinally (Table 2). The median and abaxial meristem cells in the young leaves are slightly periclinally elongated, and originate the cells of the spongy parenchyma in the mature leaves, which tend to grow isotropically. In the galls, the median cortical cells elongated anticlinally at induction, while the outer cortical cells tend to isotropy. At the stage of growth and development, both cell lineages tend to grow isotropically, but at the stages of gall maturation and senescence, the median cortical cells elongated anticlinally, while the outer cortical cells elongated periclinally (Table 2). The abaxial epidermal cells grew isotropically in the young leaves, but as they mature, these cells elongated periclinally. In the galls at the stages of induction, and growth and development, the outer epidermal cells grew isotropically, but during gall maturation and senescence, the growth is anisotropic with periclinal elongation (Table 2).

**Table 2:** Directions of cell expansion and degree of isotropy/anisotropy during the ontogenesis of *Psidium cattleianum* leaves and *Nothotrioza cattleiani* galls (μm ± standard deviation).

		Cell lineages				
Developmental stages		Adaxial/inner epidermis	Adaxial meristem/ inner cortex	Median meristem/ median cortex	Abaxial meristem/ outer cortex	Abaxial/outer epidermis
Young non-galled leaf	A	20.86 ± 0.39*d*	21.26 ± 0.33*f*	22.67 ± 0.77*d*	19.21 ± 0.36*d*	14.30 ± 0.40*c*
	P	14.00 ± 0.44**d**	17.85 ± 0.69**d**	18.24 ± 0.43**e**	15.42 ± 0.53**d**	13.10 ± 0.55**d**
	R	1.49	1.19	1.24	1.24	1.09
Mature non-galled leaf	A	21.63 ± 0.37*dc*	45.30 ± 0.92*d*	30.09 ± 0.83*d*	24.83 ± 0.59*c*	14.30 ± 0.25*c*
	P	28.06 ± 0.70**e**	20.06 ± 0.76**d**	32.30 ± 1.02**d**	27.47 ± 1.01**c**	24.77 ± 0.78**ab**
	R	0.77	2.25	0.93	0.90	0.57
Gall induction	A	20.91 ± 0.66*d*	25.72 ± 1.19*ef*	25.64 ± 1.10*d*	22.67 ± 0.68*cd*	14.62 ± 0.47*c*
	P	17.74 ± 0.79**d**	17.08 ± 0.70**d**	19.78 ± 0.87**e**	21.17 ± 1.16**cd**	16.10 ± 0.73**d**
	R	1.17	1.50	1.29	1.07	0.90
Gall growth and development	A	25.16 ± 1.10*c*	36.19 ± 1.30*c*	51.86 ± 1.92*c*	35.38 ± 1.39*b*	21.52 ± 0.81*b*
	P	33.77 ± 1.29**c**	35.46 ± 1.21**c**	47.06 ± 1.91**c**	32.59 ± 1.75**b**	20.97 ± 1.14**c**
	R	0.74	1.02	1.10	1.08	1.02
Gall maturation	A	35.16 ± 1.10*b*	52.36 ± 2.35*bd*	71.52 ± 2.51*b*	48.23 ± 2.03*a*	21.93 ± 0.59*b*
	P	42.16 ± 1.60**b**	41.54 ± 1.44**b**	53.03 ± 1.37**b**	61.25 ± 2.83**a**	24.95 ± 0.84**a**
	R	0.83	1.26	1.34	0.78	0.87
Gall senescence	A	41.70 ± 1.79*a*	84.48 ± 4.56*a*	80.75 ± 2.79*a*	48.30 ± 1.61*a*	19.09 ± 0.69*a*
	P	43.82 ± 2.03**a**	50.07 ± 1.63**a**	59.47 ± 1.59**a**	55.10 ± 1.75**a**	21.70 ± 0.74**bc**
	R	0.95	1.68	1.35	0.87	0.87

Lines labelled “A” and “P” respectively correspond to anticlinal and periclinal axes of the cells from different cell lineages at different developmental stages. Lines labelled “R” correspond to the A/P ratio from the same cell lineage at different developmental stages. Values lower than 0.9 indicate tendency to anisotropic growth with periclinal elongation; values between 0.9 and 1.1 indicate tendency to isotropic growth, and values higher than 1.1 indicate tendency to anisotropic growth with anticlinal elongation. Values followed by different italic and boldface letters in the columns indicate significant difference between anticlinal and periclinal axes of cells, respectively (ANOVA followed by t-test or multiple tests of Tukey; Kruskal-Wallis followed by Dunn's multiple tests. α = 0.05).

### Immunocytochemistry of plant cell walls

#### Non-galled leaves

**Fig 5:**
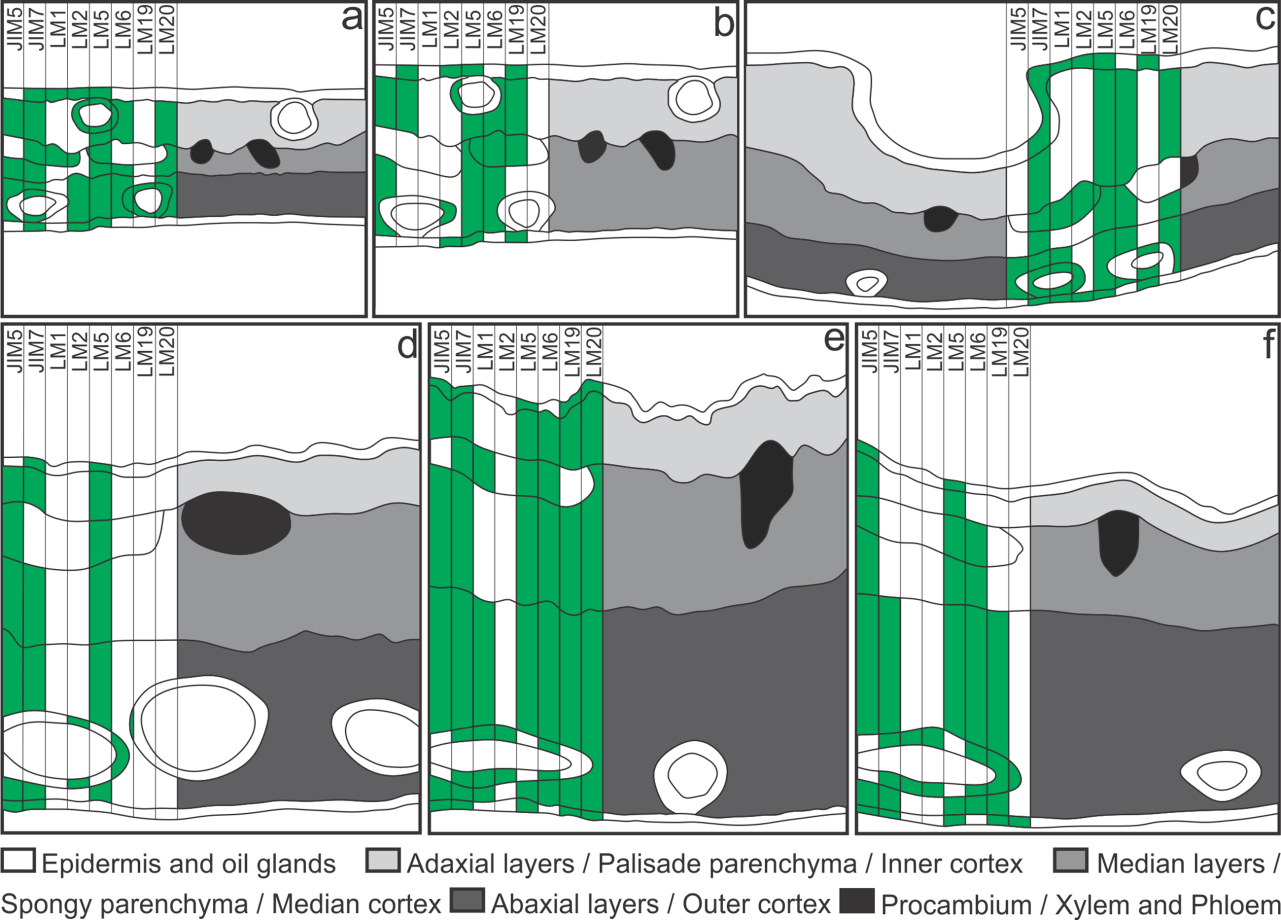
Development-dependent immunolabelling of plant cell wall pectins and proteins during the development of *Psidium cattleianum* leaves and *Nothotrioza myrtoidis* galls. Gray scale labels indicate different ontogenetic tissues and the filling in the perpendicular bars indicates positive binding of low methylesterified homogalacturonans by the JIM5 antibody, medium-high methylesterified homogalacturonans by the JIM 7 antibody, extensins by the LM1 antibody, arabinogalactan proteins by the LM2 antibody, galactans and arabinans by the LM5 and LM6 antibodies, and unesterified HGAs and high methylesterified HGAs by the LM19 and LM20 antibodies. (**a**) Young leaves, (**b**) Mature leaves, (**c-f**) Galls, (**c**) Induction, (**d**) Growth and development, (**e**) Maturation, (**f**) Senescence.

**Fig 6:**
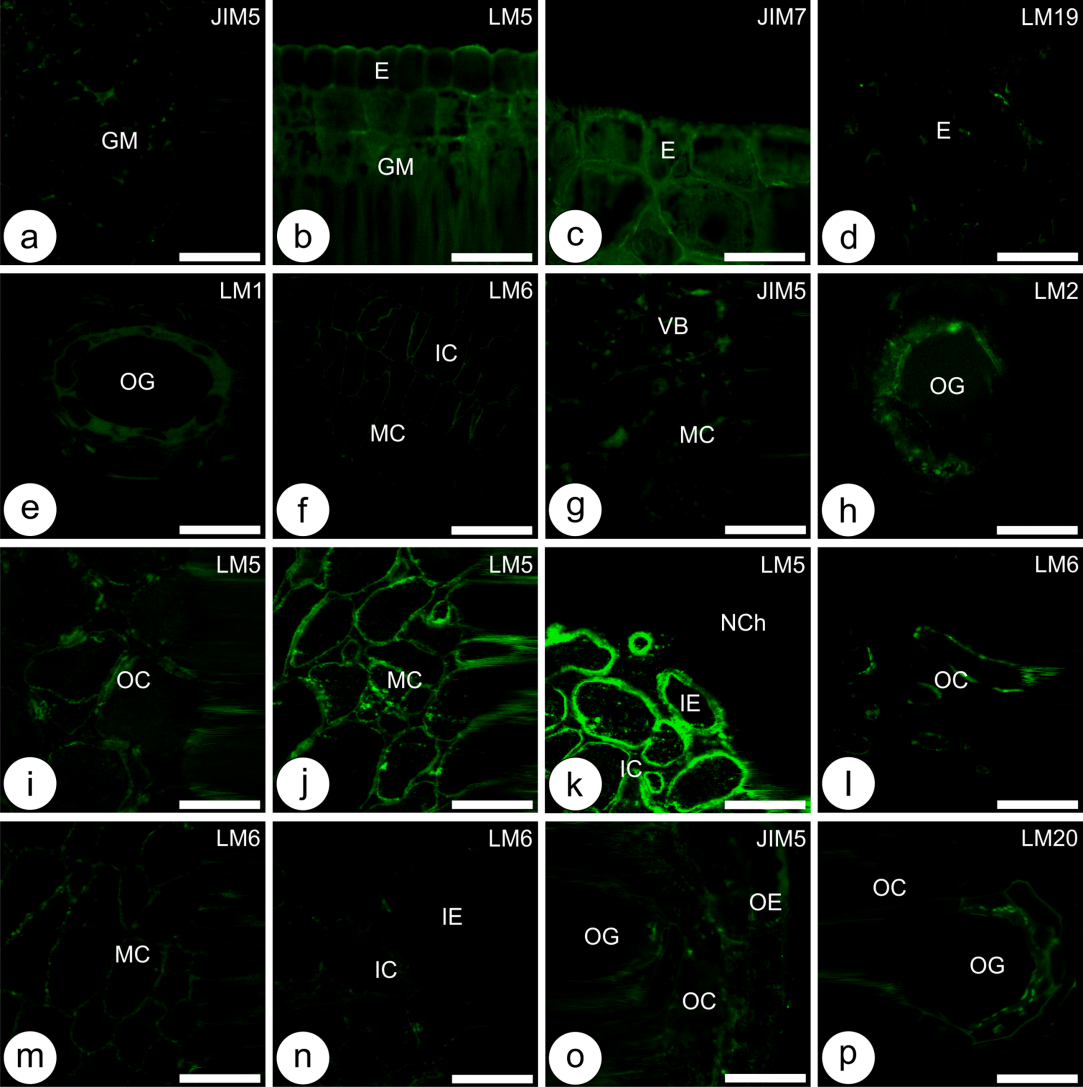
Immunocytochemistry of plant cell wall pectins and proteins during the development of non-galled leaves of *Psidium cattleianum* and galls induced by *Nothotrioza myrtoidis*. (**a, b**) Young leaves, (**c, d**) Mature leaves, (**e-p**) Galls, (**e, f**) Induction, (**g, h**) Growth and development, (**i-n**) Maturation, (**o, p**) Senescence. (**a, c, f, h-n, p**) **C**ontinuous signal of the labelling of ubiquitous epitopes, (**b, d, e, g, o**) Discontinuous signal of the labelling of microdomains of epitopes, (**a, g, o**) Low methylesterified homogalacturonans (HGAs), (**b, i-k**) Galactans, (**c**) Medium-high HGAs, (**d**) Unesterified HGAs, (**e**) Extensins, (**f, l-n**) Arabinans, (**h**) Arabinogalactan proteins, (**p**) High methylesterified HGAs. E: epidermis; GM: ground meristem; IC: inner cortex; IE: inner epidermis; MC: median cortex; NCh: nymphal chamber; OC: outer cortex; OE: Outer epidermis; OG: oil gland; VB: vascular bundle. Bars: 50 μm.

In the adaxial epidermis of young leaves, epitopes of medium-high methylesterified homogalacturonans (HGAs), extensins, arabinogalactan proteins (AGPs) and galactans are bound by the antibodies JIM7, LM1, LM2 and LM5, respectively (Fig 5a). In the abaxial surface of the epidermis, the same epitopes, except for the extensins, are bound. The cells of the oil glands have epitopes of medium-high methylesterified HGAs bound by JIM7, unesterified and high methylesterified HGAs bound by LM19 and LM20, and AGPs, galactans and arabinans bound by LM2, LM5 and LM6, respectively. In all cell layers of the chlorophyllous parenchyma, the walls have epitopes of low methylesterified HGAs bound by JIM5, high methylesterified HGAs bound both by JIM7 and LM20, and galactans and arabinans bound by LM5 and LM6, respectively. AGPs bound by LM2 are labeled strictly in the abaxial layers of the chlorophyllous parenchyma. Cells of the vascular tissues have epitopes of medium-high methylesterified HGAs, extensins, AGPs and galactans, respectively bound by JIM7, LM1, LM2 and LM5 (Fig 5a). At this developmental stage, low methylesterified HGAs (Fig 6a) widespread in the leaf tissues, extensins and AGPs in the cells of the vascular tissues are detected in microdomains of the cell walls, generating discontinuous signals. High methylesterified HGAs, AGPs, galactans (Fig 6b), arabinans, and extensins had more ubiquitous epitopes, and generated continuous signal in epidermal cells.

In mature leaves, the epidermis in the adaxial surface have epitopes of unesterified and medium-high methylesterified HGAs bound by LM19 and JIM7, and epitopes of AGPs and galactans bound by LM2 and LM5, respectively (Fig 5b). In the abaxial surface, epitopes of AGPs, galactans and unesterified HGAs were bound by LM2, LM5 and LM19, respectively. In the cells of the oil glands, only the epitopes of AGPs were bound by LM2. Cells of the chlorophyllous parenchyma have epitopes of low methylesterified HGAs, galactans and arabinans bound by JIM5, LM5 and LM6, respectively. Epitopes of unesterified and medium-high methylesterified HGAs bound by LM19 and JIM7 are strictly labeled in the palisade parenchyma. In the vascular tissues, phloem and parenchyma cells have epitopes of galactans and arabinans bound by LM5 and LM6, and unesterified HGAs bound by LM19 (Fig 5b). Cells of mature leaves have ubiquitous epitopes of low and high methylesterified HGAs (Fig 6c), galactans, and arabinans in the parenchyma and vascular tissues, which generated continuous signals. On the other hand, epitopes of AGPs, unesterified HGAs (Fig 6d), and galactans in the epidermis are more scarce and generated discontinuous signals.

#### Galls

In the galls at the stage of induction, epidermal cells of the inner and outer surfaces have epitopes of unesterified and medium-high methylesterified HGAs bound by LM19 and JIM7, extensins bound by LM1, and AGPs and galactans bound respectively by LM2 and LM5 (Fig 5c). Cells of the oil glands have the same epitopes as the epidermis, except for the absence of galactans. Epitopes of medium-high methylesterified HGAs bound by JIM7, galactans and arabinans bound respectively by LM5 and LM6, and high methylesterified HGAs bound by LM20 are detected in all gall cortical layers. Low methylesterified HGAs bound by JIM5, and extensins bound by LM1 are detected specifically in the outer cortical cell layers. Cells of the vascular bundles have epitopes of medium-high methylesterified HGAs bound by JIM7, extensins bound by LM1, and galactans bound by LM5 (Fig 5c). Cells in the galls at the stage of induction have epitopes of low methylesterified HGAs, extensins (Fig 6e), and AGPs distributed in microdomains, which generated discontinuous signals. Epitopes of high methylesterified HGAs, extensins, galactans and arabinans, and AGPs in the oil glands (Fig 6f) are more abundant and generate continuous signals.

During the stage of growth and development, the inner epidermal cells have epitopes of medium-high methylesterified HGAs bound by JIM7, and galactans bound by LM5 (Fig 5d). The outer epidermal cells have epitopes of low and medium-high methylesterified HGAs bound respectively by JIM5 and JIM7. In the cells of the oil glands, epitopes of AGPs and arabinans are bound by LM2 and LM6, respectively. All cortical cells have epitopes of low and medium-high methylesterified HGAs bound respectively by JIM5 and JIM7, and galactans bound by LM5. The epitopes bound in the cells of vascular tissues and cortices are similar, except for the absence of the epitopes of high methylesterified HGAs in the latter (Fig 5d). At this developmental stage, epitopes of low methylesterified HGAs are distributed in microdomains, which generated discontinuous signals (Fig 6g). The epitopes of medium-high methylesterified HGAs, AGPs (Fig 6h), galactans and arabinans are more abundant and generated continuous signals.

The gall inner and outer epidermal cells at the phase of maturation have epitopes of unesterified, low and high methylesterified HGAs bound by LM19, JIM5 and LM20, medium-high methylesterified HGAs bound by JIM7, and galactans bound by LM5 (Fig 5e). Cells of the oil glands have epitopes of medium-high methylesterified HGAs bound by JIM7, and epitopes of extensins, AGPs and arabinans are respectively bound by LM1, LM2 and LM6. Cells from all cortical layers have epitopes of unesterified, low, medium-high and high methylesterified HGAs bound by LM19, JIM5, JIM7 and LM20, and galactans and arabinans bound respectively by LM5 and LM6. Epitopes of extensins are labeled only in the cells from the outer cortical layers. In the cells of the vascular tissues, epitopes of medium-high methylesterified HGAs bound by JIM7, and epitopes of extensins, galactans and arabinans are respectively bound by LM1, LM5 and LM6 (Fig 5e). At the phase of maturation, the observed epitopes of unesterified HGAs and extensins were distributed in microdomains, which generated discontinuous signals. The epitopes of low and medium-high methylesterified HGAs, AGPs, galactans, and arabinans are more ubiquitous, and generated continuous signals. The galactans and arabinans are distributed in centripetal (Fig 6i–6k) and centrifugal (Fig 6l–6n) gradients in the cortical cells, respectively.

During the stage of senescence, inner epidermal cells have epitopes of low methylesterified HGAs bound by JIM5, and galactans bound by LM5 (Fig 5f). The outer epidermal cells have epitopes of unesterified, low, medium-high and high methylesterified HGAs bound by LM19, JIM5, JIM7 and LM20, and galactans bound by LM5. Epitopes of low methylesterified HGAs bound by JIM5 and galactans bound by LM5 are detected in all layers of the gall cortex. Epitopes of unesterified and medium-high methylesterified HGAs bound by LM19 and JIM7 are detected strictly in the outermost layers of the gall cortex. Epitopes of arabinans are detected by LM6 in the cells of the median and outer layers of the gall cortex. In the vascular tissues, phloem and parenchyma cells have epitopes of low methylesterified HGAs bound by JIM5, and galactans and arabinans bound respectively by LM5 and LM6 (Fig 5f). At the final stage of gall development, the epitopes of low and medium-high methylesterified HGAs are distributed in microdomains, which generate discontinuous signals (Fig 6o). Extensins, AGPs, galactans, arabinans, and unesterified HGAs (Fig 6p) are more abundant, which generate continuous signals.

## Discussion

### Ontogenetic changes in leaves lead to new cell fates in galls

The development of the simple leaves of *Psidium cattleianum* follows a common pattern described by Hara [40] for dicotyledons, in which the ground meristem precociously exhibit differentiated tissue layers, and the procambium differentiates from the median layers. This pattern has been reported for Neotropical host plants, which interact with different gall inducers [2, 10, 41, 42]. Even though such similarity is conserved across the non-galled leaves, different gall morphotypes develop, namely, the pocket gall induced by *Aceria lantanae* on *Lantana camara* [41], the bivalve-shaped gall induced by *Euphalerus ostreoides* on *Lonchocarpus muehlbergianus* [10], and the globoid galls induced by *Calophya duvauae* on *Schinus polygamus* [42] and by *Nothotrioza myrtoidis* on *Psidium myrtoides* [2].

The globoid morphotype is the most common gall shape found in the Neotropics [3], and anatomical studies on the development of such structures can elucidate the processes that lead to their morphogenesis. In fact, it has been widely reported that different taxa of insects induce morphologically distinct galls on the same host plants [10, 19, 41, 43–48], which reinforce the hypothesis that galls are the extended phenotypes of the insects [20]. From this perspective, the association of *Nothotrioza* spp. with *Psidium* spp. recently described in the Neotropics [19] represents a good model to compare if the final shapes of the galls are determined by convergent plant-dependent or divergent insect-induced characteristics. The galls induced by *N*. *myrtoidis* on *P*. *myrtoides* previously studied by Carneiro et al. [2] are globoid and extremely similar to the ones induced by *N*. *cattleiani* on *P*. *cattleianum* studied herein. Galls of *P*. *cattleianum* are induced on young leaves [18], which have reactive tissues that respond to gall induction by cell hypertrophy and determined sites of hyperplasia, leading to the formation of emergencies growing over the insect body. A similar mechanism was described for the induction of the leaf galls on *P*. *myrtoides* [2], *Piptadenia gonoacantha* [49], and *Schinus polygamus* [42], which indicates a conservative trait of galls induced on leaf surface. Cell hypertrophy and tissue hyperplasia in the median and outer cortical layers were also observed during the stage of gall growth and development. Together, such characteristics have constituted the most widespread features of insect-induced galls [50]. The stage of gall growth and development is characterized by the great increase in biomass [51], which in the case of the galls of *N*. *cattleiani* on *P*. *cattleianum* occurs via pronounced hyperplasia of the median and outer cortical cell layers, as observed for the galls of *N*. *myrtoidis* on *P*. *myrtoides* [2].

By the end of the stage of growth and development and at the beginning of maturation, the galls on *P*. *cattleianum* assume a structure commonly observed in many galls, i. e., neo-formed vascular bundles interspaced in parenchyma cells, and sclerenchyma in the outer cortical cell layers. This pattern of gall organization has been reported in the Neotropical region for the midrib galls on *Copaifera langsdorffii* [9], the bivalve-shaped gall on *Lanchocarpus muehlbergianus* [10], the intralaminar globoid galls on *Aspidosperma spruceanum* [52], and the extralaminar globoid galls on *Psidium myrtoides* [2]. Nevertheless, the discontinuous groups of sclereids found in the outer cortical layers of mature galls on *P*. *cattleianum* differ from the arrangement of a sclerenchymatic ring described by Rohfritsch [51] as pattern. The role of lignified tissues in galls is widely regarded as a defensive strategy for the gall inducers [53], which increase the adaptive value of the gall [5]. Recent studies have related developmental processes, such as lignification, to the balance of reactive oxygen species (ROS) in gall tissues [9, 10, 13, 14, 54–56]. Such subcellular signals are known to take part in plant responses to the attack by parasites and/or herbivores [57], and the oxidative stress triggered by their accumulation may affect the deposition of lignins at specific sites, as proposed by Carneiro et al. [2, 55]. The lignification of gall cortical cells by the end of the maturation stage implies the occurrence of an oxidative burst [56], which besides affecting gall structure, may constitute a signal for the beginning of senescence in galls induced by certain groups of insects.

At senescence, *N*. *cattleiani* galls exhibit spontaneous dehiscence for the escape of adults, with great similarities to the mechanism described for *N*. *myrtoidis* galls [2]. As far as we are concerned, these are the only anatomical descriptions of spontaneous mechanisms of dehiscence for insect galls. Generally, gall-inducing insects have different strategies for leaving the galls, which include actively digging a scape tunnel by the feeding larva [49, 51], when the gall tissues are completely fused. In other cases, the galls are permanently open and the inducers simply leave their galls through the pre-existing aperture, as it is observed in the leaf galls of *Pseudotectococcus rollinae* on *Rollinia laurifolia* [58], and *Callophya duvauae* on *Schinus polygamus* [42]. In the galls induced by *N*. *cattleiani* on *P*. *cattleianum*, the massive hypertrophy of the inner cortical cells during the transition from gall maturation towards senescence exerts tension on peripheral necrotic cells, which end up rupturing, setting the adult insects free. Even though the expressed phenotype is the same, i. e., the gall wall rupturing, the underlying anatomical dynamics involve peculiar and distinct cell responses in *P*. *cattleianum* galls when compared to those of *P*. *myrtoides* [2]. The growth of targeted cell lineages at very specific moments is most likely to be driven by hormones, like indol-acetic acid (IAA). Such hormone was histochemically localized in the cortical cells of the galls on *Piptadenia gonoacantha*, where phenolic substances were also accumulated [59]. This co-localization suggest that phenolics may influence cell growth due to the inhibition of the IAA oxidases [60], thus increasing the bioavailability of active IAA in the cells. The observation of phenolic inclusions in specific cell types, which grow differently, both in the galls of *P*. *myrtoides* [2, 55] and *P*. *cattleianum* corroborate this hypothesis. From the anatomical point of view, the double co-generic systems *Psidium—Nothotrioza* have conservative morphogenetical traits, but specific cell dynamics for the neo-ontogenesis [2] of flat leaves towards globoid galls.

### Dynamics of cell elongation for the neo-ontogenesis of the globoid shape

The dynamics of tissue hyperplasia and cell hypertrophy, as well as the occurrence of isotropic and/or anisotropic types of cell expansion [7] depend on the structure and functionality of cell walls [17]. Neotropical galls have been increasingly analyzed by the quantitative perspective of cell hypertrophy, with special attention to the directions of cell elongation [2, 9–12]. The tendency of shifting from isotropic to anisotropic cell elongation on *P*. *cattleianum* galls is similar to that of *Baccharis dracunculifolia* [12]. Some other galls have opposite dynamics, with cells changing from anisotropic to isotropic type of cell growth along the development, as reported for *C*. *langsdorffii* [9], *L*. *muehlbergianus* [10], and *P*. *myrtoides* galls [2]. These results suggest that the types of cell elongation in different galls are not strictly dependent on their final shapes.

The cell transformations described for the cortical parenchyma of *N*. *myrtoidis* galls ultimately generate the globoid gall morphotype by the establishment of a centrifugal gradient of cell hypertrophy [2]. Despite the macro-morphological similarity with *N*. *myrtoidis* galls, the galls induced by *N*. *cattleiani* on *P*. *cattleianum* do not have such gradient, as the median layers of the cortex are the most hypertrophic, followed by the inner and outer cortical layers. Similar patterns of cell hypertrophy were reported for the midrib galls on *C*. *langsdorffii* [9], and in the fusiform stem galls on *Marcetia taxifolia* [11]. The hypothesis of the similarity in the anatomical development of *Nothotrioza* spp. galls in *Psidium* spp. leaves is partially corroborated, as both galls exhibit time-based occurrence of tissue hyperplasia and cell hypertrophy along gall developmental stages. Nevertheless, both the sites of cell hypertrophy and the dynamics of cell elongation in the different tissue layers constitute divergent patterns between such co-generic systems.

### Immunocytochemical identity of cell lineages and cell wall functionalities

The dynamic properties of cell walls and tissue lineages regulate developmental processes in plants [17]. In the case of the galls induced by *Nothotrioza* spp. on *Psidium* spp., which are both globoid but somewhat anatomically different, the pectin and protein composition of the cell walls vary according to the cell lineages. Epidermal cells of *P*. *cattleianum* leaves and *N*. *cattleiani* galls remain uniseriate during their ontogenesis, and exhibit conservative cell wall composition. The association of HGAs and galactans seems to be especially important for the identity of epidermal cells in this gall, since their epitopes are constant throughout leaf and gall development. The detection of galactans, alone or associated to HGAs, is an indicative of cell wall rigidity [61, 62], and has been associated to sites of hyperplasia [63]. In fact, galactans and HGAs detection in epidermal cell walls of leaves and galls confer them stability and rigidity. These two properties are reinforced by the detection of extensins [64, 65] in the epidermal cells of young non-galled leaves and galls of *P*. *cattleianum* at the induction stage. The reinforcement of young epidermal cells by the extensins reflects the expected pattern of epidermal cells differentiation, which divide anticlinally to accompany the growth of either leaves or galls, without hypertrophying or losing the uniseriate organization. Current results corroborate the functional roles of HGAs and galactans in fast-dividing cells as observed by Xu et al. [63] in *Musa* tissues.

Oil glands, which are specialized epidermal cells, have different chemical composition in their cell walls along the development of leaves and galls. In addition to HGAs, arabinans, galactans, and extensins, oil glands have AGPs, which are believed to play a role in the prevention of programmed cell death [66, 67]. Also, they modulate cell division and expansion in all developmental stages, as previously reported by Carneiro et al. [2] in the galls induced by *N*. *myrtoidis* on *P*. *myrtoides*. The AGPs seem to be markers of the secretory cells identity, as they were also detected in the mucilage cells of *Araucaria angustifolia* [68], in the secretory ducts of galls on *B*. *reticularia* [13]. The modulation of oil gland cell properties relies on the balance among flexibility, rigidity and porosity. The flexibility is conferred by arabinans to expanding cells [63], while the rigidity is the result of the stabilization by the cross bonds established by extensins among cell wall polymers [33, 64, 65]. Porosity is conferred by the association of arabinans and HGAs due to the depletion of calcium-mediated cross links [38]. The chemical composition in the walls of ordinary epidermal cells and oil gland cells are maintained for the effective mediation of the gall—environment interface, as far as protection of gall tissues and gall inducer is concerned. Both the chemical composition of the cell walls and the localization of the oil glands reveal a similar defensive phenotype of galls [2] in the double co-generic systems *Psidium—Nothotrioza*.

The chemical identity of almost all cortical parenchyma cell walls retains some similarity to that of epidermal cells in terms of the presence of HGAs and galactans. Nevertheless, the association of these epitopes with arabinans enhances the flexibility and porosity of their cell walls [38] due to the alignment of HGAs, as previously proposed by Foster et al. [69]. These functional aspects of parenchyma cell walls guarantee the establishment of metabolic gradients in the cortex, as histochemically detected for other galls [9, 54, 55]. Porosity seems to be a key property of the host plant cell walls, which is conservative for galls. In current model, it is especially useful for parenchyma cells, which demand higher cell to cell flux of molecules. This property was reported by Oliveira et al. [56] in *Baccharis dracunculifolia* galls and by Carneiro et al. [2] in *Psidium myrtoides* galls, where the porosity is related to the reallocation of molecules drained from the senescent galls towards the leaves. Besides porosity, the co-occurrence of a centripetal gradient of galactans and a centrifugal gradient of arabinans in mature galls should maintain the inner cortical cells more rigid [61, 62] and the outer cortical cells more flexible [63]. Nevertheless, the enhanced rigidity of the outer cortex is guaranteed by lignin deposition in the thick secondary cell walls, instead of by the presence of galactans. In fact, histometry (*cf*. Table 1) validates that the flexible arabinan-rich cells of the outer cortex, prior to lignifying, hypertrophy more than the rigid galactan-rich cells of the inner cortex, corroborating the functionality of such epitopes.

Changes on the cellular identities of epidermal and parenchyma lineages are ultimately related to variations on the degree of methylesterification of the HGAs, the major pectins of the cell walls. Demethylesterified HGAs cross link to calcium [70], a well-known cellular signaler that integrates the symplast and apoplast for many cell responses [71], and rigidify cell walls. Current analyses reveal that cycles of high methylesterified HGAs production are interposed by cycles of demethylesterification by pectin methylesterases (PMEs) to create a balance between such forms. Up to gall maturation, the degree of HGAs methylesterification does not corroborate the gradient of rigidity assumed for the gall cortical layers. At gall senescence, however there is a tendency of increased rigidity due to lower degree of pectin methylesterification, more pronouncedly in the outer cortex. In fact, the action of PMEs during the morphogenesis of galls has been assumed to occur at the late stages of gall development, when the tissues are more rigid and the degree of cell hypertrophy is the highest [2, 14]. In *N*. *cattleiani* galls, cell hypertrophy continues to occur until senescence in the median and inner cortices. This is in accordance with the balance on the degree of pectin methylesterification, and plays crucial role in the cellular mechanism of gall dehiscence.

Similar fluctuations on the epitopes related to cell wall rigidity were observed in the vascular system, and in its less plastic tissues. Structural integrity was maintained in the vascular bundles for the effective nutrition of the gall inducer, which is a phloem feeder [72]. Furthermore, the vascular tissues of senescent galls have cell wall epitopes similar to that of mature leaves, which is the standard morphogenetical pattern. For all other cell lineages, the comparison of cell wall epitopes in mature leaves and mature galls corroborate that galls represent the neo-ontogenesis of leaf tissues [2], i. e., divergent cell fates. The neo-ontogenesis is most evident in the cells of the ground system, which undergo hypertrophy and hyperplasia, the core processes by which galls are anatomically defined. The fine regulation of such processes, either related to convergent or divergent final forms of the galls, are peculiarities of each system. Herein, the immunocytochemical analyses in the galls of the double co-generic systems *Psidium—Nothotrioza* reveal completely different set of cell wall epitopes. These differences reveal fine regulation of cell processes towards unique functional-structural aspects required for gall development. Current comparative approach corroborates the hypothesis that the extended phenotype concept [20] can be validated at the cellular and subcellular levels, which are true insect-induced unique characteristics.
